# Malaria Infection and Risk for Endemic Burkitt Lymphoma: A Systematic Review and Meta-Analysis

**DOI:** 10.3390/ijerph18115886

**Published:** 2021-05-30

**Authors:** Kwuntida Uthaisar Kotepui, Manas Kotepui

**Affiliations:** Medical Technology Program, School of Allied Health Sciences, Walailak University, Tha Sala, Nakhon Si Thammarat 80160, Thailand; kwuntida.ut@wu.ac.th

**Keywords:** endemic Burkitt lymphoma, eBL, *Plasmodium*, malaria

## Abstract

*Background*: Malaria infection is reportedly linked to endemic Burkitt lymphoma (eBL) in malaria-endemic areas. This study aimed to pool the overall risk (or odds) of eBL among children with previous or concurrent malaria infection. *Methods*: We searched PubMed, Web of Science, Scopus, and reference lists of publications for potentially relevant studies on malaria infection and eBL. The quality of the included studies was assessed using the Joanna Briggs Institute for case-control studies. Random-effects meta-analysis was used to summarize whether the odds of eBL can be increased by (1) malaria infection or (2) elevated titer of IgGs to malaria antigen. The level of heterogeneity was evaluated using Cochran’s Q statistic and I^2^. The individual study data, pooled odds, and confidence interval (CI) were illustrated using the forest plot. Publication bias was assessed using funnel plots and Egger’s test. *Results:* Ten studies were included, reporting the number of malaria cases in eBL and non-eBL (5 studied malaria infection and the odds of eBL; five studied the burden of IgGs to malarial antigens and the odds of eBL). According to the meta-analysis results, the odds of eBL was not increased by malaria infection (*p* = 0.562, OR: 0.87, 95% CI: 0.54–1.39, I^2^: 93.5%, malaria in eBL: 604/1506 cases, malaria in non-eBL: 2117/4549 cases) and the elevated titer of IgGs to malaria antigen (*p* = 0.051, OR: 1.50, 95% CI: 1.00–2.25, I^2^: 89%, increased IgG titer in eBL: 1059/1736 cases, increased IgG titer in non-eBL: 847/1722 cases). In meta-regression analysis, sex was not a confounding factor for the effect size of malaria infection and eBL (*p* = 0.10) and that of increased IgGs and eBL (*p* = 0.80). *Conclusions*: Malaria infection and IgG titer elevation did not increase the risk for eBL among children. However, the included studies, which are only few, do not generally agree on this point. Therefore, the risk for eBL in children diagnosed with malaria should be investigated further by longitudinal studies to confirm our evidence-based approach.

## 1. Background

Malaria is caused by one of the six *Plasmodium* spp. including *P. falciparum*, *P. vivax*, *P. malariae*, *P. ovale curtisi*, *P. ovale wallikeri*, and *P. knowlesi* [1]. Malaria and its mortality mostly occur in children aged below 5 years from 31 African countries [2]. The short-term effects of malaria infection are classified into uncomplicated malaria and severe malaria. Severe malaria is caused mainly by *P. falciparum* and seldom by *P. vivax*, *P. malariae*, *P. ovale curtisi*, *P. ovale wallikeri*, or *P. knowlesi* [3,4,5,6]. Although the short-term effects of malaria infection are well described, the long-term effects, such as risk of tumor or cancer, remain poorly understood.

Endemic Burkitt lymphoma (eBL) is an aggressive non-Hodgkin lymphoma originating from B-cell precursors [7]. It is the most common childhood cancer in African children and is mostly located in the face (maxilla or mandible) or in the abdomen [8]. Previous epidemiological reports in malaria-endemic areas suggested that malaria infection is linked to eBL risk in children [9,10,11,12]. The risk factors for eBL development might be increased in African populations who are exposed to *P. falciparum* [13]. Nevertheless, most of the studies suggesting the link between malaria infection and eBL are case-control studies; therefore, the study design and confounders might have some bias. In a most recent cohort study, malaria increased the risk of non-Hodgkin and Hodgkin lymphomas (hazard ratio (HR): 3.49, 95% confidence interval (CI): 1.42–8.56) but did not increase the risk of other lymphoid neoplasms [14]. To our knowledge, no meta-analysis has assessed the risk of eBL or other cancers. Therefore, the present study aimed to pool the overall risk (or odds) of developing eBL in children with previous or concurrent malaria infection. The results of this study will further guide the longitudinal research on the risks of developing malaria infection and eBL in large population cohorts.

## 2. Methods

### 2.1. Protocol

The systematic review followed the Preferred Reporting Items for Systematic Reviews and Meta-Analyses guidelines [15].

### 2.2. Search Strategy and Eligibility Criteria

We searched for studies on malaria infection and Burkitt lymphoma in PubMed, Web of Science, Scopus, and reference lists of articles published between January 1975 and 28 January 2021. In the literature search, we combined the following search terms: “(malaria OR Plasmodium) AND (neoplasia OR neoplasias OR neoplasm OR tumors OR tumor OR cancer OR cancers OR malignancy OR malignancies) AND (risk OR risks OR odd OR odds),” as provided in Table S1. We enrolled those studies that were full original research in peer-reviewed journals without language or publication data restriction. To assure that all relevant articles in the literature were reviewed, we further searched and reviewed the reference lists of the enrolled articles. The inclusion criteria were as follows: (1) studies reporting the prevalence or incidence of malaria infection and EBL, and (2) studies that included data on the following outcomes of interest: number of malaria infection cases in eBL, number of malaria infection cases in non-eBL or control groups, the burden of IgGs to malarial antigens in eBL. Conversely, we used the following exclusion criteria: studies reporting Epstein–Barr virus (EBV)/herpesvirus and malaria infection, studies in which data on the outcome of interest could not be extracted, in vitro studies, case reports or case series, mathematical, or statistical models of eBL or malaria. For studies with the same group of participants and published in more than one journal, the most comprehensive report was included.

### 2.3. Study Selection

In selecting relevant studies, we began by screening the titles and abstracts retrieved from the three abovementioned electronic databases. The potentially relevant studies were full-text-screened using the inclusion and exclusion criteria. Any disagreement in the study selection was resolved by discussion for consensus.

### 2.4. Data Extraction

We independently extracted data of interest including the first author name, publication year, study sites, study period, study design, age range, sex, participants, number of malaria cases in case/control groups, the burden of IgGs to malarial antigens in eBL. Any disagreements during study extraction were resolved through discussion for consensus.

### 2.5. Risk-of-Bias Assessment

We assessed the quality of all included studies using the Joanna Briggs Institute for case-control studies [16]. Each study was assessed as yes, no, unclear, or not applicable on 10 checklists. The total score of 10 was totaled to generate the overall quality score, which ranged from 0 to 10 (Table S2). Then, the studies were classified into (1) having a high (<35%), (2) moderate (35–75%), or low (>75%) risk-of-bias. Disagreements in the risk assessment were resolved through discussion for consensus.

### 2.6. Outcomes

The primary outcome was the malaria infection and odds of eBL. The secondary outcome was the increased titer of IgGs to malarial antigens and odds of eBL.

### 2.7. Data Analysis

Random-effects meta-analysis was used to summarize (1) the association between malaria infection and the odds of eBL, and (2) the association between the increased titer of IgGs to malarial antigens and the odds of eBL. The level of heterogeneity among the odds of each study was assessed using Cochran’s Q statistic and I^2^. The individual study outcome, pooled odds, and CIs were displayed using the forest plot. Publication bias was evaluated using the funnel plots and Egger’s test. Possible sources of heterogeneity and possible confounding factors affecting the effect size were explored by meta-regression and subgroup analysis. In the meta-regression analysis, the relationship between age or sex and the pooled odds were examined. In the subgroup analysis, the study site or patients’ characteristics was used to determine the difference in each subgroup and within-group heterogeneity. Statistical data were analyzed using Stata version 14.2 (Stata Corporation, College Station, TX, USA).

## 3. Results

### 3.1. Search Results

**Figure 1:**
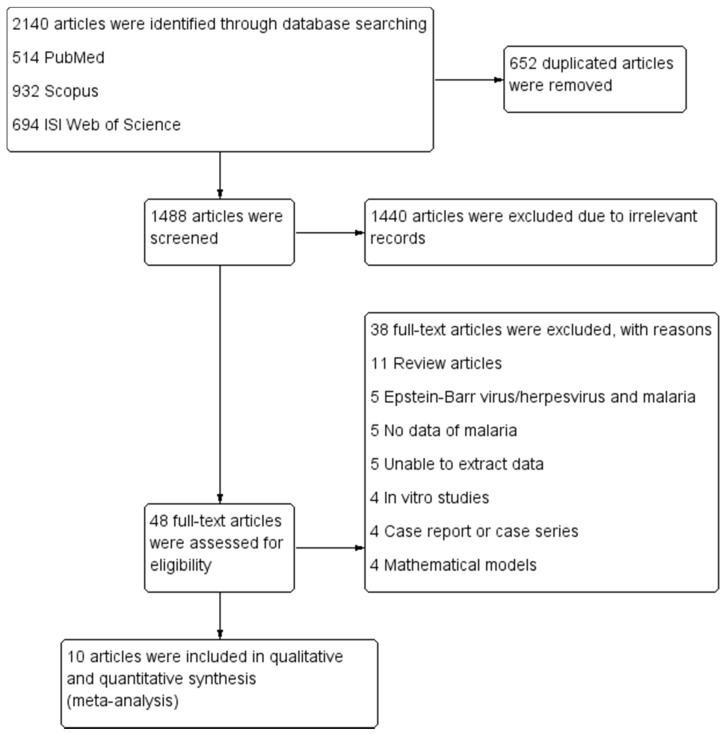
Flowchart showing the selection of eligible articles.

We identified 2140 articles through PubMed (514 articles), Scopus (932 articles), and ISI Web of Science (694 articles). After 652 duplicated articles were removed, 1488 remaining articles were screened for titles and abstracts, and then 1440 nonrelated articles were excluded. Subsequently, we examined the full text of the 48 remaining articles and excluded 38 articles for the following reasons: 11 were review articles, 5 were articles reporting EBV/herpesvirus and malaria, 5 had no data of malaria, 5 had data that could not be extracted, 4 were in vitro studies, 4 were case reports and series, and 4 articles were mathematical models. Ultimately, 10 articles were included in the qualitative and quantitative syntheses (meta-analysis) (Figure 1).

### 3.2. Characteristics and Quality of the Included Studies

Table 1 and Table 2 summarize the characteristics of the included studies. Of the 10 studies, five [9,10,11,12,14] reported the number of malaria cases in eBL/non-eBL (Table 1), and the other five [17,18,19,20,21] reported the burden of IgGs to malarial antigens among eBL/non-eBL (Table 2). Five studies that reported the number of malaria cases in eBL/non-eBL were conducted in Uganda [9,11], Ghana [9], Malawi [10], Tanzania and Kenya [12], and Sweden [14]. Four of which [9,10,11,12] were case-control studies investigating children aged 0–15 years, and the other one [14] was a cohort study that followed up participants who developed lymphoid neoplasm (mean age: 34.7 years). The five other studies reporting the burden of IgGs to malarial antigens in eBL/non-eBL were case-control studies investigating participants aged 0–15 years and were conducted in Malawi [17,21], Ghana [18,20], and Uganda [19]. As shown in Table S2, the quality of all of the included studies was moderate (>75%).

### 3.3. Malaria Infection and Odds of eBL

**Figure 2:**
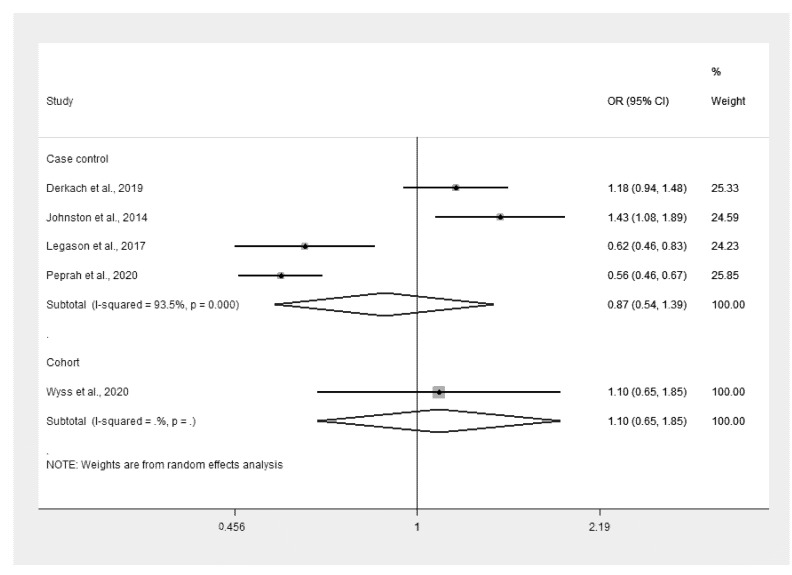
Malaria infection and odds of eBL. Note: Wyss et al.’s study has no p-value because it is the only study in the cohort subgroup; the *p*-value of 0.0000 reported for the section on case-control studies was interpreted as *p* < 0.0001. % Weighted: the impact proportion of each study to the pooled effect. Black dot symbol on the black horizontal line: the point estimate for each study. Black horizontal line: CI. White diamond symbol: pooled OR in each subgroup. Solid line in the middle of the graph at 1: no difference in OR between the two groups. CI: confidence interval, OR: odds ratio.

The malaria infection and odds of eBL were estimated using four studies [9,10,11,12]. The study conducted in Malawi in 2005–2010 by Johnston et al. [10] and another study conducted in Ghana (1965–1994) and Uganda (2010–2015) by Derkach et al. [9] showed that malaria infection increased the odds of eBL. Conversely, two studies [11,12] conducted in Uganda, Tanzania, and Kenya showed that malaria infection decreased the odds of eBL. Overall, the results showed that malaria infection did not increase the odds of eBL (*p* = 0.562, OR: 0.87, 95% CI: 0.54–1.39, I^2^: 93.5%, malaria infection in eBL: 604/1506, malaria infection in non-eBL: 2117/4549) (Figure 2). Meanwhile, a cohort study in Sweden in 1987–2015 showed that malaria infection did increase the risk of Burkitt lymphoma [14].

### 3.4. Increased Titer of IgGs to Malarial Antigens and Odds of eBL

**Figure 3:**
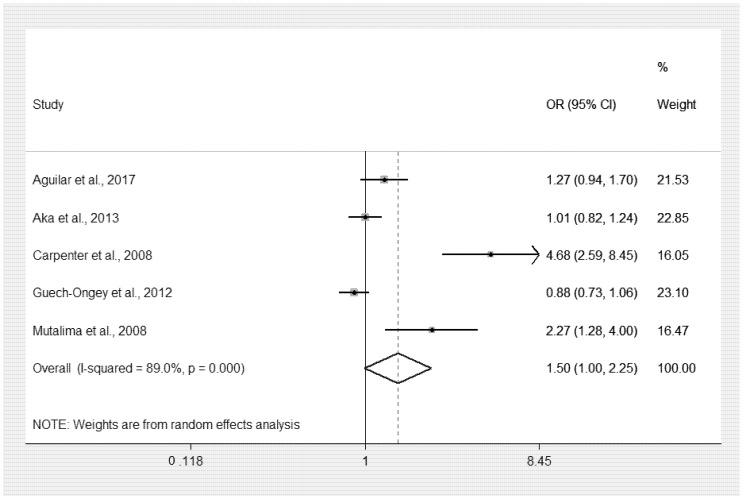
Increased titer of IgGs and odds of eBL. Note: The *p*-value of 0.0000 reported for the section on case-control studies was interpreted as *p* < 0.0001. % Weighted: the impact proportion of each study to the pooled effect, Black dot symbol on black horizontal line: point estimate for each study, Black horizontal line: CI, White diamond symbol: pooled OR, Solid line in the middle of the graph at 1: no difference in the OR between the two groups. CI: confidence interval, OR: odds ratio.

The increased titer of IgGs to malarial antigens and odds of eBL were estimated using five studies [17,18,19,20,21]. Two studies conducted in Uganda [19] and Malawi [21] showed that the elevated titer of IgGs to malaria antigen increased the odds of eBL. In contrast, three studies [17,18,20] showed that the odds of eBL was not increased by the elevated titer of IgGs to malaria antigen. Overall, the pooled analysis showed that the increased titer of IgGs to malaria antigen did increase the odds of eBL (*p* = 0.051, OR: 1.50, 95% CI: 1.00–2.25, I^2^: 89%, increased titer of IgGs in eBL (1059/1736), increased titer of IgGs in non-eBL (847/1722) (Figure 3).

### 3.5. Meta-Regression Analysis

**Figure 4:**
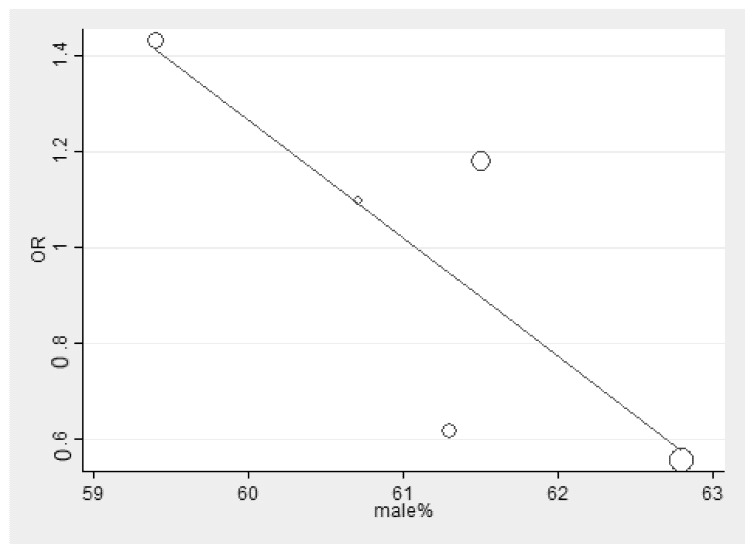
Meta-regression analysis of sex and effect size (OR) of malaria infection and eBL. OR: odds ratio. Increased in male % could predict the lower OR of malaria infection and eBL.

**Figure 5:**
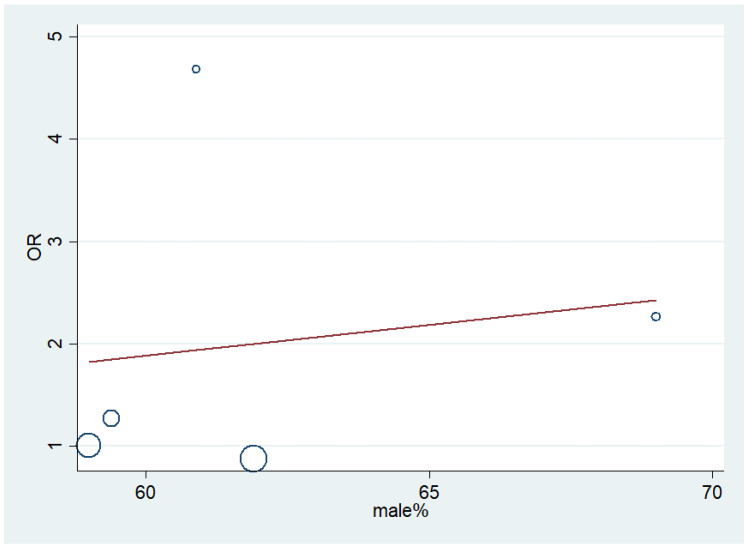
Meta-regression analysis of sex and effect size (OR) of titer of IgGs and eBL. OR: odds ratio. Increased in male % could predict the higher OR of titer of IgGs and eBL.

To determine whether sex confounded the effect size (pooled odds of malaria infection and eBL, or the pooled odds of increased titer of IgGs to malarial antigens and eBL), we conducted meta-regression analysis. The results showed that sex did not confound the effect size between the pooled odds of malaria infection and eBL (*p* = 0.10, coefficient: −0.25, standard error: 0.10) (Figure 4). In addition, sex did not confound the effect size between the pooled odds of the increased titer of IgGs to malarial antigens and eBL (*p* = 0.80, coefficient: 0.06, standard error: 0.22) (Figure 5).

### 3.6. Publication Bias

Given that the number of the included studies per one outcome was less than 10, the publication bias among the included studies could not be assessed [22].

## 4. Discussion

All case-control studies included in this study were conducted in African regions. Although the meta-analysis demonstrated no risk of eBL among children infected with malaria because of the heterogeneity of the included studies (93.5%), the results of the individual study should be considered. For example, the study conducted by Johnston et al. [10] in Malawi in 2005–2010 showed an increase in the odds of eBL, while two studies [11,12] conducted in Uganda, Tanzania, and Kenya showed a decrease in the odds of eBL among children with malaria. Lastly, Derkach et al. [9] in Ghana and Uganda did find a correlation between malaria infection and the risk or odds of eBL. The prevalence, density, or genetic diversity of *P. falciparum* might be the triggering factors of malaria exposure or co-factors for endemic eBL in children [19,21,23,24]. In addition, the risk of developing eBL is elevated in people with low maternal income and paternal education [12]; these factors are well-known contributing factors for higher malaria intensity [25], suggesting to be another mechanism of eBL risk. The risk for eBL may also increase with immunity to malaria infection. Inpatient malaria treatment is reportedly associated with a higher risk for eBL among children [12]; most of the malaria treatment is required in younger children than in older adults because younger children lacks immunity against malaria. Multiple episodes of *P. falciparum* infection can stimulate B cells, which enhance the translocation of the *Myc* proto-oncogene on human chromosome 8 in eBL tumorigenesis [26]. Alternatively, these multiple episodes can increase the risk for eBL indirectly through T-cell immunity impairment, leading to uncontrolled EBV proliferation [27].

The present meta-analysis demonstrated that the increased IgG titer to malaria antigen did not increase the risk for eBL. However, the included studies had high heterogeneity (89%). Interestingly, the studies conducted by Carpenter et al. [19] in Uganda and Mutalima et al. [21] in Malawi showed that the increased IgG titer to malaria antigen increased the risk for eBL. Meanwhile, three studies [17,18,20] conducted in Malawi and Ghana showed no increase in the risk of eBL among patients who had an elevated IgG titer to malaria antigen. According to a previous study, the odds ratio for eBL increased as the EBV antibodies and the number of malaria treatment episodes increased, suggesting that EBV and malaria may act together to increase the risk for eBL in children [19]. The EBV viral load seems to increase in children who live in malaria-hyperendemic areas than those in malaria-hypoendemic areas [28]. The mechanism of synergism of EBV and malaria antibody to increase the risk for eBL was postulated to be the malaria-causing reactivation of memory B cells, which are latently infected with EBV, and/or suppression of EBV-specific T-cell immunity [27,29,30].

However, the present study has limitations. First, the number of included studies was limited because of the few available research published in the electronic databases. Although we additionally searched the reference lists of review articles, we found no relevant articles. Second, the high level of heterogeneity of the included studies in the meta-analysis indicated that the pooled analysis should be carefully interpreted by the reader. Third, the case-control studies, which were highly influenced by the selection bias and confounders, were selected by authors; therefore, the outcome bias might have occurred. Fourth, both case-control and cohort studies were used in the pooled analysis of malaria infection and odds of eBL; thus, the result of the subgroup or individual study should be interpreted.

## 5. Conclusions

In conclusion, the meta-analysis demonstrated the risk for eBL was not different among patients with malaria infection. In addition, the increased titer of IgGs to malaria antigen did not increase the risk for eBL. To support our evidence-based approach, we need to conduct additional longitudinal studies investigating the risk for eBL among children living in malaria-endemic areas.

## Figures and Tables

**Table 1:** Malaria infection and risk of Burkitt lymphoma.

No.	Author, Year	Study Area (Years of Survey)	Study Design	Age (mean ± SD or Median [Range])	Sex (male [%])	Participants	Percentage of Malaria Cases in Case/Control Groups
1.	Derkach et al., 2019	Ghana (1965–1994) Uganda (2010–2015)	Case-control study	0–15 years All cases Cases Range (n): 0–2 (9), 3–5 (56), 6–8 (110), 9–11 (96), 12–14 (62), ≥15 (11) Controls Range (n): 0–2 (38), 3–5 (99), 6–8 (216), 9–11 (234), 12–14 (142), ≥15 (20) Ghana Cases Range (n): 0–2 (2), 3–5 (29), 6–8 (62), 9–11 (36), 12–14 (13), ≥15 (18) Controls Range (n): 0–2 (5), 3–5 26), 6–8 (54), 9–11 (43), 12–14 (13), ≥15 (8) Uganda Cases Range (n): 0–2 (7), 3–5 27), 6–8 (48), 9–11 (60), 12–14 (49), ≥15 (3) Controls Range (n): 0–2 (33), 3–5 (73), 6–8 (162), 9–11 (191), 12–14 (129), ≥15 (12)	All Cases (341): male (61.5%) Controls (749): male (56.6%) Ghana Cases (150): male (63.3%) Controls (149): male (63.8%) Uganda: Cases (191): male (60.2%) Controls (600): male (54.8%)	Cases: Burkitt lymphoma Controls: healthy children in the same age range	All: cases (49.9%), controls (42.3%) Ghana: cases (69.1%), controls (31.3%) Uganda: cases (35.1%), controls (45%)
2.	Johnston et al., 2014	Malawi (2005–2010)	Case-control study	0–15 years Cases: 7.7 (se 0.2) Controls: 6.5 (se 0.3)	Cases (303): male (59.4%) Controls (274): male (55.8%)	Cases: Burkitt lymphoma Controls: children admitted to the same hospital with a wide range of both malignant and nonmalignant conditions	Cases (64.7%), controls (45.3%)
3.	Legason et al., 2017	Uganda (2011–2015)	Case-control study	0–15 years Cases: 7.7 (3–28) Controls: 7.34 (3–40)	Cases (199): male (61.3%) Controls (624): male (55.1%)	Cases: Burkitt lymphoma Controls: healthy children in the same age range	Cases (34.7%), controls (56.3%)
4.	Peprah et al., 2020	Uganda, Tanzania, and Kenya (2010–2016)	Case-control study	0–15 years All cases Cases (689): 7.3 ± 3.7 Controls (2934): 7.5 ± 3.5 Uganda Cases (316): 8.0 ± 3.4 Controls (1150): 7.7 ± 3.3 Tanzania: Cases (71): 6.8 ± 3.8 Controls (819): 7.4 ± 3.3 Kenya: Cases (246): 6.6 ± 3.8 Case controls (965): 7.4 ± 3.7	All cases Cases (689): male (62.8%) Controls (2934): male (53.1) Uganda Cases (316): male (62%) Controls (1150): male (53%) Tanzania: Cases (127): male (55.9%) Controls (819): male (52.7%) Kenya: Cases (246): male (67.5%) Controls (965): male (53.6%)	Cases: Burkitt lymphoma Controls: health facility, school, or neighborhood controls	All: cases (25.4%), controls (45.7%) Uganda; cases (34.6%), controls (51%) Tanzania: cases (19.5%), controls (41.3%) Kenya: cases (15.8%), controls (43%)
5.	Wyss et al., 2020	Sweden (1987–2015)	Cohort study	Incidence of lymphoid neoplasm (4125): 34.7 ± 18.5 Matched comparator (66,997): NA	Incidence of lymphoid neoplasm (4125): male (60.7%) Matched comparator (66,997): male (59.7%)	Cases: Lymphoid neoplasm Controls: matched comparator cohort from the general population	Incidence of lymphoid neoplasm (0.36%), matched comparator (0.33%)

**Table 2:** Burkitt lymphoma for the burden of IgGs to malarial antigens.

No.	Author, Year	Study Area (Years of the Survey)	Study Design	Age Range (Years)	Sex (Male [%])	Participants	Burden of IgGs to Malarial Antigens
1.	Aguilar et al., 2017	Malawi (2005–2006)	Case-control study	Cases: 7.8 ± 2.9 Controls: 6.3 ± 3.7	Cases (271): male (59.4%) Controls (174): male (56.9%)	Cases: Burkitt lymphoma Controls: histologically diagnosed with other conditions	Cases (271): lower (15.5%), medium (46.1%), higher (38.4%) Controls (171): lower (33.9%), medium (33.9%), higher (32.2%)
2.	Aka et al., 2013	Ghana (1965–1994)	Case-control study	0–15 years	Cases (354): male (59%) Controls (384): male (55.7%)	Cases: Burkitt lymphoma Controls: healthy location-matched controls	Cases (354): positive ≥ 1 of 4 antigens (95.5%), negative to all antigens (4.52%) Controls (384): positive ≥ 1 of 4 antigens (94.8%), negative to all antigens (5.21%)
3.	Carpenter et al., 2008	Uganda (1994–1999)	Case-control study	0–15 years	Cases (325): male (60.9%) Controls (579): male (39.1%)	Cases: Burkitt lymphoma Controls: HIV-negative children aged 15 years or less with benign, noninfectious, surgical, or orthopedic conditions	Cases (325): high (12.9%), low (22.2%), negative (3.69%) Controls (579): high (2.76%), low (6.56%), negative (2.76%)
4.	Guech-Ongey et al., 2012	Ghana (1965–1994)	Case-control study	0–14 years	Cases (657): male (61.9%) Controls (498): male (54.4%)	Cases: Burkitt lymphoma Controls: healthy children from the same community where the case arose	Cases (657): lower (41.2%), medium (33%), higher (25.6%) Controls (498): lower (33.1%), medium (33.7%), higher (33.1%)
5.	Mutalima et al., 2008	Malawi (2005–2006)	Case-control study	0–15 years	Cases (148): male (60.1%) Controls (104): male (54.8%)	Cases: Burkitt lymphoma Controls: histologically diagnosed with other conditions	Cases (129): high/medium (50.4%), low/negative (8.5%) Controls (90): high/medium (22.2%), low/negative (27.8%)

## Data Availability

All data related to this study was available in the main manuscript and supplementary files.

## Supplementary Materials

The following are available online at https://www.mdpi.com/article/10.3390/ijerph18115886/s1, Table S1: Search terms, Table S2: Quality of the included studies.
