# Supplementary material for: Malaria Infection and Risk for Endemic Burkitt Lymphoma: A Systematic Review and Meta-Analysis
Source: Int J Environ Res Public Health. 2021 May 30;18(11):5886. doi: 10.3390/ijerph18115886 (PMC8198990; doi:10.3390/ijerph18115886)
Supplement: Supplementary file 1 [file ijerph-18-05886-s001.zip › ijerph-1230620-supplementary.pdf]

Table S1. Search terms.

Databases	Search terms	Search date
MEDLINE (PubMed)	(malaria OR Plasmodium) AND (neoplasia OR neoplasias OR neoplasm OR tumors OR tumor OR cancer OR cancers OR malignancy OR malignancies) AND (risk OR risks OR odd OR odds)<br><br>Search option: All fields<br>Search results: 514	27 March 2021
Scopus	(malaria OR Plasmodium) AND (neoplasia OR neoplasias OR neoplasm OR tumors OR tumor OR cancer OR cancers OR malignancy OR malignancies) AND (risk OR risks OR odd OR odds)<br><br>Search option: title, abstract, keywords<br>Search results: 932	27 March 2021
ISI Web of Science	(malaria OR Plasmodium) AND (neoplasia OR neoplasias OR neoplasm OR tumors OR tumor OR cancer OR cancers OR malignancy OR malignancies) AND (risk OR risks OR odd OR odds)<br><br>Search option: All fields<br>Search results: 694	27 March 2021

Table S2. Assessment of risk of bias relating to individual studies using the Joanna Briggs Institute for case-control studies.

No.	Authors	Groups comparable	Cases and controls matched	Same criteria used for identification of cases and controls	Exposure measured in a standard, valid and reliable way	Exposure measured in the same way for cases and controls	Confounding factors identified	Strategies to deal with confounding factors	Outcomes assessed in a standard, valid and reliable way	The exposure period of interest long enough	Appropriate statistical analysis	Score (10)
1	Derkach et al., 2019	Yes	Yes	Yes	Yes	Yes	Unclear	Unclear	Yes	Not applicable	Yes	7 (70%)
2	Johnston et al., 2014	Yes	Yes	Yes	Yes	Yes	Unclear	Unclear	Yes	Not applicable	Yes	7 (70%)
3	Legason et al., 2017	Yes	Yes	Yes	Yes	Yes	Unclear	Unclear	Yes	Not applicable	Yes	7 (70%)
4	Peprah et al., 2020	Yes	Yes	Yes	Yes	Yes	Unclear	Unclear	Yes	Not applicable	Yes	7 (70%)
5	Wyss et al., 2020	Yes	Yes	Yes	Yes	Yes	Unclear	Unclear	Yes	Not applicable	Yes	7 (70%)
6	Aguilar et al., 2017	Yes	Yes	Yes	Yes	Yes	Unclear	Unclear	Yes	Not applicable	Yes	7 (70%)
7	Aka et al., 2013	Yes	Yes	Yes	Yes	Yes	Unclear	Unclear	Yes	Not applicable	Yes	7 (70%)
8	Carpenter et al., 2008	Yes	Yes	Yes	Yes	Yes	Unclear	Unclear	Yes	Not applicable	Yes	7 (70%)
9	Guech-Ongey et al., 2012	Yes	Yes	Yes	Yes	Yes	Unclear	Unclear	Yes	Not applicable	Yes	7 (70%)
10	Mutalima et al., 2008	Yes	Yes	Yes	Yes	Yes	Unclear	Unclear	Yes	Not applicable	Yes	7 (70%)
